# Faster vegetative growth in *Arabidopsis thaliana* in heat and drought

**DOI:** 10.17912/micropub.biology.001892

**Published:** 2026-02-11

**Authors:** Basia Love, Jeffrey K Conner, Emily B Josephs, Sophia F Buysse

**Affiliations:** 1 Plant Biology Department, Michigan State University, East Lansing, Michigan, United States; 2 Ecology, Evolution, and Behavior Program, Michigan State University, East Lansing, Michigan, United States; 3 Kellogg Biological Station, Hickory Corners, Michigan, United States; 4 Plant Resilience Institute, Michigan State University, East Lansing, Michigan, United States

## Abstract

Climate change is increasing average temperature and decreasing water availability, both stressors that affect plant growth. High heat consistently decreases growth rate but plants may grow rapidly to escape drought or grow more slowly and avoid water loss. We use *Arabidopsis thaliana* to investigate how heat and drought together shape plant growth and if their impact differs between plants that escape or avoid drought. Our results show that leaf area growth differs but the response to heat and drought is consistent between locally adapted populations in widespread plants like *A. thaliana.*

**Figure 1. Leaf area growth differs by treatment and population while leaf number only differs by treatment:**
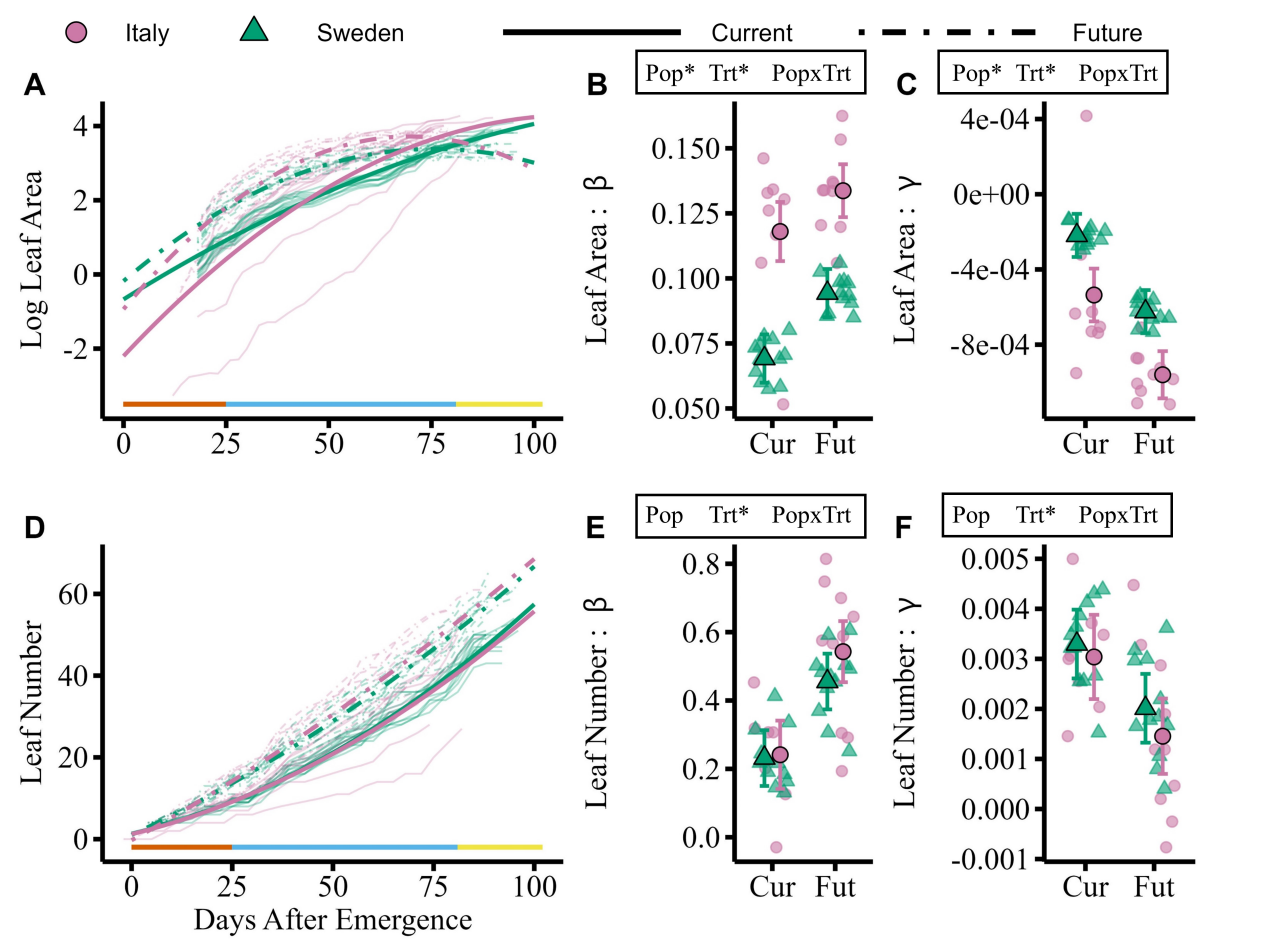
Growth curves (A, D) generated from estimated marginal means of population differentiation in slope, β (B, E), and curvature, γ (C, F), for log transformed rosette leaf area (A, B, C) and untransformed rosette leaf number (D, E, F). In panels A and D, bold lines are model predicted growth curves while faint lines are raw growth for each genotype. The solid line along the x axis indicates chamber conditions by season assuming a plant emerged three days after planting; left to right these are fall (orange), winter (blue) and spring (yellow). In panels B, C, E, and F, smaller points represent the β or γ of each genotype and large points are the estimated marginal means with 95% confidence intervals. Model predictors above panels indicate p-value; asterisks denote *p* < 0.001 and all others are *p* > 0.25.

## Description

Rapidly rising global temperatures in the last 50 years have increased heat waves and droughts, stressing plants and animals (Seleiman et al., 2021). Plant drought responses vary based on the severity, timing, and duration of drought. For example, drought at the end of the growing season can favor the evolution of escape: rapid vegetative growth, earlier flowering, and higher photosynthetic capacity that allows a plant to complete reproduction before the onset of drought (Kooyers, 2015). Mild and long-term droughts can favor drought avoidance: an increase in root-to-shoot ratio and corresponding decrease in stomatal conductance and growth rate that preserve internal water content, avoiding water deficit inside the leaf (Kooyers, 2015). Similarly, plants may respond to heat through faster flowering and decreased growth among other responses that can be additive or antagonistic when heat and drought are experienced together (Gao et al., 2020; Stewart et al., 2016; Vile et al., 2012). Here, we manipulated heat and drought to investigate genetic differentiation for vegetative growth rate and plasticity of growth rate in a projected hot and dry future climate between two locally adapted populations of *Arabidopsis thaliana.*

Vegetative growth is ecologically important because larger leaf area allows for increased photosynthesis but can also cause greater water loss during drought. In *A. thaliana,* drought decreases leaf area growth rate and leaf area at maturity, but the magnitude varies across populations (Bac-Molenaar et al., 2016; Bouchabke et al., 2008; Clauw et al., 2016). The impact of heat and drought on growth is less clear. Relative growth rate of *A. thaliana* from 15 populations across Europe in hot (23°C), well-watered conditions is statistically independent from climate of origin (Fletcher et al., 2022). However, an additional study with 249 genotypes found that populations of *A. thaliana* from colder climates grow more slowly in cool (16°C) and cold ( 6°C) temperatures when under mild drought (Clauw et al., 2022). This inconsistency across studies suggests that the nuances of heat and drought treatments matter for experimental outcomes. Studies applying realistic simulations of likely future climates, including manipulation of multiple abiotic stressors in tandem, are needed to make accurate predictions about population responses to climate change (Zandalinas & Mittler, 2022).

We studied growth of two locally adapted populations of *A. thaliana* from Italy and Sweden (Ågren & Schemske, 2012) in a chamber common garden experiment. The timing of drought in the native range of *A. thaliana* ranges from mild, short droughts during the growing season to an intense, terminal drought at the end of the growing season and this variation corresponds with within-species variation in drought avoidance and drought escape (Monroe et al., 2018). Our populations span this gradient. The Italian population is adapted to a shorter growing season with a terminal drought (Mojica et al., 2016) which may select for fast growth. Conversely, the Swedish population is adapted to more frequent, shorter droughts during a long growing season (Mojica et al., 2016) which may select for slow growth.

We used two common gardens as our treatments; one treatment simulates the average field conditions in the fall and spring at the Swedish seed collection site while the other is both 4°C hotter and consistently drier, simulating projected future climates. Prior work shows that the Italian population escapes drought while the Sweden population avoids drought (Buysse et al., 2026; Mojica et al., 2016) but these studies did not measure vegetative growth rate.

We measured rosette leaf area starting 15 days after planting until each plant bolted ( Fig. 1A ). In both populations, plants in the Future treatment increased leaf area quickly when young and achieved a maximum leaf area earlier than in the Current treatment. In both treatments, the Italian population grew faster ( Fig. 1B ) with growth slowing as plants neared bolting (i.e., more negative curvature; Fig. 1C ). Both linear and nonlinear growth differ between treatments and populations, but there is no population by treatment interaction, indicating both populations respond to the Future treatment similarly.

We counted rosette leaf number from plant emergence through bolting ( Fig. 1D ). Leaf number is positively correlated with leaf area ( *r* = 0.857, *p* <0.001). Plants in the Future treatment produced leaves more quickly than in those in the Current treatment ( Fig. 1E ) but the Current treatment plants increased the rate of leaf production near bolting more than the plants in the Future treatment ( Fig. 1F ). Leaf number only differed between treatments; there was no significant differentiation between populations or a population by treatment interaction in either the linear or nonlinear components.

Overall, we found that the Italian and Swedish populations have differences in the rate of leaf area expansion but similar growth through leaf number. This may be from differences in leaf size; individual Italian leaves are larger in both treatments and fewer leaves are needed to reach the maximum leaf area measured from top-down photos. The observation that leaf area growth decreases (negative quadratic) but leaf number growth increases (positive quadratic) over time likely results from leaf overlap reducing overall leaf area growth rates despite continued leaf production. Faster leaf area growth in the Italian population ( Fig. 1A, B) is consistent with drought escape to maximize photosynthesis while conditions are favorable. Further, the Italian population bolted earlier than the Swedish population in both treatments (population mean ± standard error Days to Bolting in Italy (Current) = 78.5 ± 1.1; Italy (Future) = 79.2 ± 1.0; Sweden (Current) = 88.5 ± 0.9; Sweden (Future) = 89.3 ± 0.9), also consistent with drought escape. The slower leaf area growth and later bolting in the Swedish population is consistent with drought avoidance where investment in stress protecting pathways slows vegetative growth. While we attribute differences in leaf area growth to drought strategy, heat is a contributing factor as the same pattern was observed between these populations in a well-watered, high temperature experiment (Stewart et al., 2016).

The increased growth in both leaf number and leaf area in the Future treatment indicates plasticity to escape drought (Kooyers, 2015). This result differs from previous studies where heat and drought individually decreased leaf area and leaf area growth across *A. thaliana* early in the life cycle (Bac-Molenaar et al., 2016; Bouchabke et al., 2008; Clauw et al., 2015; Gao et al., 2020) and heat and drought together decreased leaf number at flowering (Vile et al., 2012). The disagreement in the direction of plasticity in response to heat and/or drought could be due to each study using different genotypes of *A. thaliana.* However, since our populations from the extremes of the European range showed the same plasticity, differences in treatments between studies likely caused the difference in plasticity. While many studies quantify growth in or after heat and drought, the length of treatments, degree of stress, and timing of growth measurements vary widely.

## Methods

*Growth Conditions*

We grew 12 native genotypes from each of two populations: Castelnuovo di Porto, Italy and Rödåsen, Sweden in two chamber common gardens. There is likely minimal genetic variation between genotypes within each population because *A. thaliana* is primarily self-fertilizing and prior unpublished studies identified little within-population variation in flowering time, freezing tolerance, and fitness (referenced in Ågren & Schemske, 2012). Seeds were collected from separate maternal plants in the field in Sweden in 2002 and Italy in 2003; these genotypes have not been confirmed via sequencing as unique but more recent collections from the Swedish site have not detected any identical genotypes (Jon Ågren, personal communication, January 2026). We grew field collected seeds in chamber common gardens for at least one generation to remove parental effects caused by environmental variation between the two field sites. We then stratified chamber collected seeds in 0.15% agarose gel in the dark at 4°C for 5 days before planting five seeds of the same genotype per pot in 150g Lamberts General Propagation mix watered to soil holding capacity (i.e., 100% soil moisture). We prepared ten additional pots that were oven dried at 60°C for 7 days to determine 0% soil moisture. We grew one pot per genotype in each of two common gardens in BioChambers growth chambers at Michigan State University ( *n* = 12 genotypes x 2 populations x 2 treatments = 48 total plants). The “Current” common garden simulated the current climate near the Sweden seed collection site and the “Future” simulated a hotter and drier environment based on climate projections for the next 50 years if global temperature rises by 2°C. We thinned plants to one plant per pot after four weeks of growth, keeping the plant centered in the pot. Six Italian genotypes had no emergence (four in Current, two in Future).

We determined Current treatment temperatures using data from Weather Underground (weatherunderground.com) for the Sundsvall-Timra Airport (49km from the Sweden field collection site) spanning 1998 - 2020. We split temperatures from August through October into four subsets and used the median day and night temperature of each subset as the chamber temperature for one week to simulate fall when plants are emerging in the field (Ågren & Schemske, 2012; Table 1). Global temperature rise will have a larger impact in Sweden, so the Future treatment was consistently 4°C hotter than the Current treatment (IPCC, 2021). By October, the mean temperature in Sweden is below 10°C, which is the minimum temperature for our chambers. Thus, for both treatments, we used 10°C during the day and 6°C overnight for 8 weeks to simulate winter (November – April in the field). To simulate spring, we used the median day and night temperature for one week in the field starting in April as the chamber temperature for one week and continued chronologically until all plants bolted. The Future treatment was returned to 4°C hotter than the Current treatment. Daylengths were determined with the same method as temperature except that we used a subset method during the winter simulation (Table 1).

We maintained soil moisture at 45% in the Current treatment and 20% in the Future treatment by weight. We weighed pots daily during the simulated fall and three times a week during the simulated winter. In the simulated spring, we weighed pots every other day. On the intermediate day, we weighed a subset of plants in each treatment and watered all plants in that treatment with the average amount of water needed by the subset.

To reduce microclimate variation, we rotated plants around each chamber two times a week.

*Phenotyping*

We captured growth rate through rosette leaf number and leaf area. We recorded leaf number twice a week once a plant had two leaves through bolting except for week 8. We quantified leaf area starting in week 5 through bolting. We took photos on a cell phone camera set to 1x zoom on a tripod two feet above the plants three times a week. While some leaves overlap, the top-down view provided an accurate view of the leaf area that natural sunlight would hit. We measured leaf area of each plant using polygons in ImageJ (Schneider et al., 2012). Plants bolted between week 11 and 15. At bolting, plants transition from vegetative to reproductive growth and we stopped our measurements.

*Statistical Analysis*

We ran linear models using *lm()* in R (v.4.4.1) to determine differences in growth measured by leaf area (log10 transformed) and leaf number (untransformed). We used a two-step process to account for non-independence of repeated measures on the same plant over time. In the first step, we fit each individual plant with a quadratic curve to describe the growth over time for each measurement quantified by the slope (beta) and curvature (gamma). These quadratic curves visually match the growth of each plant well (comparison plots available at the GitHub link below). In step two, we used population, treatment, and their interaction as predictor variables in four separate models where the response variable was the beta (β) and gamma (γ) terms generated in step one: leaf area β, leaf area γ, leaf number β, or leaf number γ (Italy Current *n* = 8; Italy Future *n* = 10; Sweden Current *n* = 12; Sweden Future *n* = 12 for each model). This allowed us to interpret genetic differentiation for growth (population), plastic changes (treatment), and genetic differentiation for plasticity in growth (interaction). By fitting each genotype separately in the first step, we do not need to account for variation in sample sizes for dates after plants bolted and were removed from our experiment. However, this method underestimates uncertainty because the confidence intervals around the linear (β) and nonlinear (γ) growth parameters estimated in step one are not incorporated in step two.

To analyze days to bolting, we used a linear model identical to step two described above with days between bolting and emergence as the response variable and population, treatment, and their interaction as predictor variables. Estimated marginal means from the *emmeans* (Lenth, 2024) package are reported.

Plots were made with *emmeans* , *ggplot2* and *ggpubr* (Kassambara, 2023; Lenth, 2024; Wickham, 2016) *.* All data, chamber settings, and code are available at https://github.com/sfbuysse/arabidopsis-SW-IT-growth .

**Table 1.** Growth chamber conditions for our Current and Future treatments. PAR was maintained at 140 in both treatments. Temperatures (°C) are written as day temperature:night temperature. The Current treatment was continuously maintained at 45% soil moisture and the Future treatment at 20% soil moisture, including during the simulated winter.

**Table:** 

	Simulating in Sweden	Current Temperature	Future Temperature	Daylength
Week 1	Fall	19:14	23:18	16h45m
Week 2	Fall	15:12	19:16	15h15m
Week 3	Fall	13:9	17:13	13h30m
Week 4	Fall	10:7	14:11	12h15m
Week 5	Winter	10:6	10:6	10h15m
Week 6	Winter	10:6	10:6	7h30m
Week 7	Winter	10:6	10:6	5h30m
Week 8	Winter	10:6	10:6	5h15m
Week 9	Winter	10:6	10:6	7h15m
Week 10	Winter	10:6	10:6	8h45m
Week 11	Winter	10:6	10:6	12h45m
Week 12	Winter	10:6	10:6	15h15m
Week 13	May 4 – 10	10:6	14:10	17h0m
Week 14	May 11 – 17	10:6	14:10	17h45m
Week 15	May 18 - 24	10:6	14:10	18h30m
